# A Rare Case of Metastatic Primary Peritoneal Ependymoma: A Case Report and Literature Review

**DOI:** 10.1155/2020/9805847

**Published:** 2020-08-14

**Authors:** Ben Man Fei Cheung, Johnny Kin Sang Lau, Anthony W. I. Lo, Mai-Yee Luk, Kwok Keung Yuen

**Affiliations:** ^1^Department of Clinical Oncology, Queen Mary Hospital, Hong Kong; ^2^Anatomical Pathology Division, Queen Mary Hospital, Hong Kong

## Abstract

**Background:**

Primary peritoneal ependymoma is an exceedingly rare tumour with only four cases reported in the literature. It typically follows an indolent disease course. We describe a rare case of metastatic primary peritoneal ependymoma which was treated with chemotherapy and radiotherapy resulting in prolonged survival to date for 10 years. *Case Presentation*. The patient was a 23-year-old female on presentation. She presented with right upper quadrant pain associated with an abdominal mass. Computed tomography demonstrated a large mass displacing the liver. Debulking surgery was done revealing a tumour arising from the peritoneum as well as multiple metastatic pleural and peritoneal nodules. Pathology was consistent with primary peritoneal ependymoma. The patient was then treated with multiple lines of chemotherapy containing etoposide as the backbone. She also received palliative radiotherapy to the thoracic metastases with good and durable response.

**Conclusion:**

We reported a rare case of metastatic primary peritoneal ependymoma. Etoposide containing the chemotherapy regimen is effective in the treatment of peritoneal ependymoma. Radiotherapy is also effective for palliation of local symptoms with durable response.

## 1. Background

Ependymomas are rare tumours arising from the ependymal cells of the central nervous system. It typically develops intracranially for paediatric population and in the spine for the adult population [1]. Although extraneural metastasis of CNS ependymoma is well documented [2], primary peritoneal ependymoma is an exceedingly rare condition with only a few cases reported to date [3]. This poses a unique clinical challenge when selecting optimal treatment. We present a case of metastatic primary peritoneal ependymoma treated with chemotherapy and radiotherapy that resulted in prolonged survival.

## 2. Case Presentation

### 2.1. History

**Figure 1:**
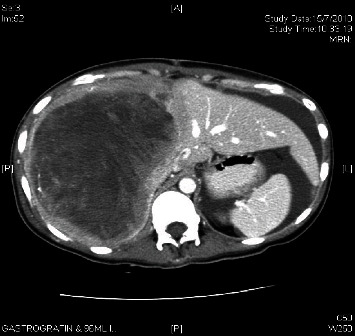
Preoperative imaging demonstrating a large tumour displacing the liver.

We present a case of metastatic primary peritoneal ependymoma treated with multiple lines of chemotherapy and radiotherapy. The patient, a 23-year-old female, initially presented with right upper quadrant pain associated with a palpable mass. Computed tomography (CT) demonstrated a large tumour measuring 17.5 cm × 15.9 cm displacing the liver (Figure 1). Debulking surgery was done, revealing a large tumour arising from the peritoneum compressing on the liver and multiple peritoneal nodules, as well as right metastatic pleural nodules. No ovarian or pelvic lesions were detected during the operation. The main tumour and peritoneal nodules were excised.

### 2.2. Pathology

Macroscopically, the peritoneal nodule was circumscribed with a firm to soft consistency. The tumour had a fleshy cut surface and was tan in colour. Haemorrhage and necrosis were not seen.

**Figure 2:**
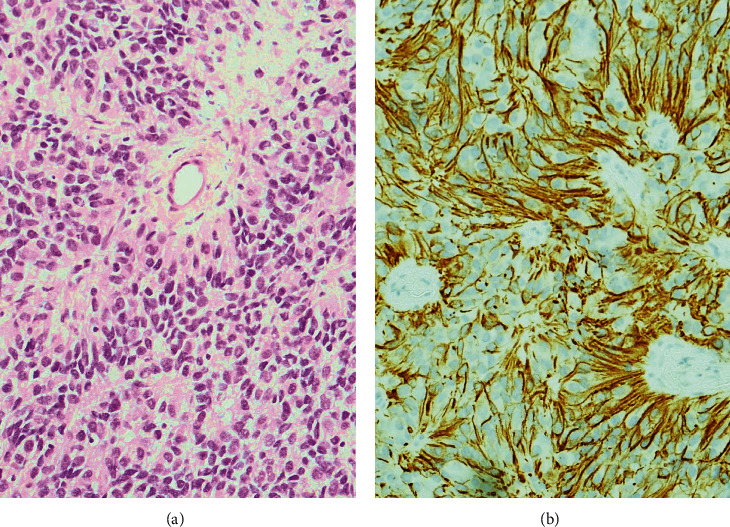
(a) H&E section of the tumour. (b) Tumour cells stained with glial fibrillary acid protein.

The tumour was cellular consisting of elongated tumour cells with oval, mildly pleomorphic nuclei (Figure 2). Pseudorosettes, consisting of tumour cells palisading around fibrovascular cores, were seen. True rosettes with tumour cells arranged around a fibrillary stroma were also noted. Mitotic figures were rare (Figure 2(a), H&E section, ×100). These tumour cells were immunoreactive to glial fibrillary acid protein (GFAP, Figure 2(b), ×200). The tumour cells were also immunoreactive to EMA with a cytoplasmic and paranuclear dot-like pattern.

Proliferation index Ki67 was less than 1%.

Ependymoma was suspected at this juncture. Differential diagnoses included metastatic tumour from the CNS ependymoma, metastatic monoepidermal teratomas from the ovaries, ovarian stromal cell tumours, GIST, and small blue round cell tumours such as Ewing sarcoma or neuroblastoma. Further staining was done to rule out these alternative diagnoses. The tumour was negative for c-KIT, inhibin, chromogranin, and synaptophysin. In view of the rosette configuration, Ewing sarcoma should be ruled out. The tumour was negative for Fli1. It was also negative for both the EWSR1 gene rearrangement by break-apart FISH and the RT-PCR test for the chimeric transcript EWSR1-Fli1.

MRI brain and whole spine were done to rule out metastatic CNS ependymoma, which was negative. No ovarian tumours were noted in other imaging investigations. As no CNS lesion and ovarian lesions were present, the patient was diagnosed with primary peritoneal ependymoma.

### 2.3. Initial Systemic Treatment

The patient later developed rapid progression of the pleural nodules after surgery leading to chest wall pain and shortness of breath. CT revealed multiple metastases in the thorax. She was given oral etoposide (50 mg/day 3 weeks on, 1 week off) for 1 year with partial response. Her shortness of breath and chest wall pain also improved significantly with chemotherapy.

Upon disease progression after 1 year of etoposide, the patient was given paclitaxel/carboplatin (TC) for 10 cycles with partial response. Chemotherapy was stopped after 10 cycles. However, after 4 months of cessation, the disease progressed. The patient was then treated with cyclophosphamide but developed disease progression again within 2 months. At this juncture, the patient's right lung was already extensively infiltrated with tumour. The patient was subsequently treated with etoposide/ifosfamide/cisplatin (VIP) for 6 cycles with partial response and good palliation of symptoms. The patient had drug holiday after completing 6 cycles of VIP.

### 2.4. Palliative Radiotherapy

**Figure 3:**
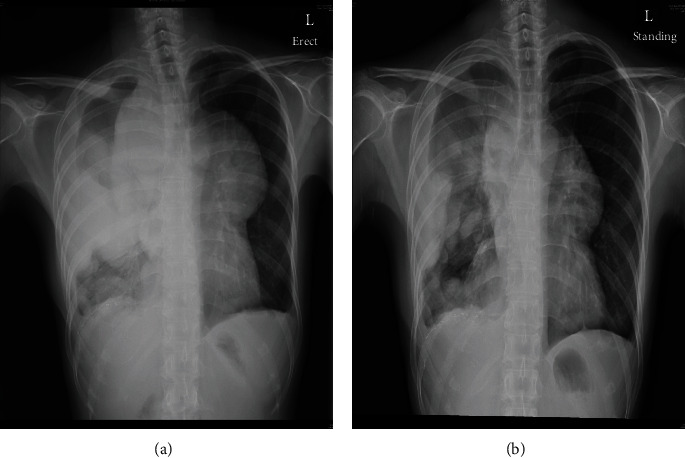
(a) Chest radiograph before thoracic radiotherapy. (b) Chest radiograph 4 months after thoracic radiotherapy.

After 4 months of drug holiday, the right lung tumour progressed markedly, leading to compression of the right main bronchus and mediastinum. The patient also developed significant shortness of breath, requiring high-flow oxygen therapy. 28 Gray in 7 daily fractions of palliative radiotherapy was given to the mediastinum and right lung tumour. The patient was treated supine using 10-megavolt photon with an anterior posterior opposing beam arrangement. The radiotherapy was planned using conventional technique due to the urgency of treatment, and the tumour was clearly delineated on chest radiograph. The radiotherapy led to a marked reduction in tumour size and improvement in symptoms within 4 months after the initial radiotherapy (Figure 3). Durability of response was 3 years.

### 2.5. Further Systemic Therapy

Oral etoposide was later rechallenged after radiotherapy. This led to a prolonged treatment response of around 5 years with very slow progression. The patient is still doing well to date for 10 years since diagnosis.

## 3. Discussion

### 3.1. Overview of Extraneural Ependymomas

Ependymoma of the nervous system originates from the ependymal cells lining the nervous tissue. Apart from CNS ependymoma, a rare distinct disease entity, namely, extraneural ependymoma has been documented. Although extraneural ependymoma is rare, occurrences of ovarian, mediastinal, sacrococcygeal, and peritoneal ependymoma have nonetheless been described [4–7]. Extraneural ependymoma can be classified into sacrococcygeal, pelvic, and extrapelvic according to its anatomical position.

The most common extraneural ependymomas are found in the subcutaneous sacrococcygeal and presacral space [8]. The most common histological subtype is myxopapillary which resembles its CNS counterpart. It was suggested that it was derived from ectopic nervous tissue from adjacent ependymal rests or extradural filum terminale [5]. This hypothesis was also supported by the fact that sacrococcygeal ependymomas resemble CNS ependymomas histologically. It is often locally aggressive with soft tissue and bony invasion leading to nerve compression or bone destruction [9]. Distant metastases have also been documented for up to 20% of patients [8]. The lung was the most common site for metastasis; other sites such as lymph nodes and pleura were described as well [10–13]. The mainstay of treatment would be resection of the tumour if possible. Radiotherapy and chemotherapy were not demonstrated to be efficacious in the treatment of sacrococcygeal ependymoma [8, 13].

Pelvic and extrapelvic ependymomas represent a distinct subtype of extraneural ependymoma. It is exclusively present for women. Pelvic ependymomas were reported to originate from the ovary, broad ligament, and mesovarium [4, 14–17]. Ovarian ependymomas can either present as pure ependymoma or as a component of a teratoma [4]. There has been evidence that extraneural ependymoma especially those originating from the ovaries can be of germ cell origin [18].

Extrapelvic ependymomas were reported in the peritoneum, mediastinum, lungs, and liver [6, 7, 19–21]. Extrapelvic ependymoma represents the rarest entity of all extraneural ependymomas with 10 cases in the mediastinum, 4 cases from the peritoneum, and 1 case in the lungs and liver, respectively. Mediastinal ependymoma is the most common subcategory which typically originates from the posterior mediastinum [6, 22]. Multiple pathogenic mechanisms were proposed for extrapelvic ependymoma due to the multiplicity of possible sites. Due to the proximity of the posterior mediastinum to the CNS, it is likely that mediastinal ependymoma originates from ectopic ependymal rest [22]. Alternatively, it was suggested that neometaplasia of peritoneal tissue can give rise to peritoneal ependymoma [4].

Extrasacrococcygeal ependymomas usually behaves indolently and are typically slow growing [6]. The mainstay of treatment is resection of disease. Following surgery, the tumour may recur locally but it rarely metastasizes [7, 14, 15]. Chemotherapy, hormonal therapy, and radiotherapy have been used to treat extrasacrococcygeal ependymomas with varying levels of success [3, 14, 15, 23].

### 3.2. Overview of Peritoneal Ependymomas

Primary peritoneal ependymomas are exceedingly rare. They are classified as extrapelvic ependymoma. Only 4 cases (including the present case) have been documented in the literature to the best of our knowledge [3, 7, 21]. Clinically, all cases presented with local symptoms such as abdominal pain or incidental finding of an abdominal mass. Pathologically, the reported cases demonstrated classical features of ependymoma. All reported cases showed perivascular pseudorosettes which are tumour cells arranged radially around a fibrovascular core. Necrosis is typically absent with a low mitotic figure except for one case which demonstrated anaplastic features with high mitotic features and necrosis [3]. GFAP was strongly positive in all cases.

Despite the small amount of reported cases, the natural history and aggressiveness of the disease were quite variable. All patients were treated with surgical resection initially. Verdun and Owen reported a case of peritoneal ependymoma presented with a right upper quadrant mass and multiple intra-abdominal tumours. The patient received surgery initially but later developed recurrence 2 years and 24 years after the initial surgery [7]. All local recurrences were treated with surgery.

Yamamoto et al. reported a case of peritoneal ependymoma with peritoneal and multiple pelvic masses on presentation. The patient received debulking surgery with miliary peritoneal lesion remaining in the peritoneal pouch of Douglas [21]. No adjuvant therapy was given. During the 10-month follow-up, there was no further progression of the tumour.

Finally, Mogler et al. reported a case of anaplastic peritoneal ependymoma which was treated with etoposide/carboplatin and multiple debulking surgeries. The disease was well controlled with chemotherapy but quickly rebound after stopping chemotherapy, thus requiring further debulking surgery.

All reported cases had multiple intra-abdominal masses on diagnosis with a larger index abdominal mass as well as multiple peritoneal masses [3, 7, 21]. As such, patients often required multiple debulking surgeries as a complete resection of disease was quite challenging. This may be a potential cause of the high rate of local recurrence. Interestingly, despite dissemination within the abdominal cavity in other cases, our case was the first case with distant metastasis.

The clinical details of reported cases of peritoneal ependymoma are summarized in Table 1.

### 3.3. Review of Treatment Options

Our patient's disease follows a much more aggressive course with rapid disease progression and metastasis. Interestingly, the histopathology was indolent and provided no clue to possible aggressive behaviours. It also produced significant local symptoms which could only be controlled through chemotherapy and radiotherapy. Owing to the rarity of the disease, it poses a clinical challenge for the choice of optimal therapy. Some success was derived from treating ovarian ependymoma with chemotherapy and irradiation from scarce case reports.

### 3.4. Chemotherapy in Recurrent/Metastatic Neural Ependymomas

Chemotherapy was occasionally employed in patients with recurrent or metastatic intracranial or neural ependymomas; as clinical experience is more mature in this regard, the treatment regimen can be extrapolated from it.

Chamberlain reported that etoposide is efficacious in recurrent neural ependymoma [24]. Trials demonstrated that it is both effective and well tolerated in recurrent brain tumours including ependymoma even in paediatric population [25, 26]. Other agents such as paclitaxel, platinum, or ifosfamide also had activity against recurrent ependymoma [27–29].

### 3.5. Systemic Therapy in Extraneural Ependymomas

A wide array of systemic therapy has been attempted for recurrent extraneural ependymomas. More clinical experience was available for pelvic ependymomas compared to other extraneural ependymomas in terms of systemic therapy.

Etoposide was one of the agents that was repeatedly employed with good response in various case reports. Mikami et al. reported a case of ovarian ependymoma treated with a prolonged course of oral etoposide [14]. Combination chemotherapy has also been employed with success. Hirahara et al. reported a case of pure ovarian ependymoma which had undergone chemotherapy such as cisplatin, CAP (cisplatin/aclarubicin/cyclophosphamide), VAC (vincristine/dactinomycin/cyclophosphamide), EP (etoposide/cisplatin), and doxorubicin/mitomycin C [15]. Hino et al. reported a case of ovarian ependymoma failing BEP (bleomycin/etoposide/cisplatin) with complete response after treatment with TIP (paclitaxel/ifosfamide/cisplatin) [30].

Apart from chemotherapy, hormonal therapy has also been used in the literature as extraneural ependymoma often expresses oestrogen and progesterone receptors [23, 31]. As such, aromatase inhibitors and GHRH analogue were used in the treatment in ovarian ependymoma.

As for cases of extrapelvic ependymomas, Mogler et al. reported the use of etoposide/carboplatin in a case of anaplastic peritoneal ependymoma resulting in stabilization of disease which rebounded after stopping [3]. Ye et al. reported a case of mediastinal ependymoma receiving adjuvant temozolomide following surgery and RT for neck recurrence [22]. No other use of chemotherapy was documented for other cases of extrapelvic ependymomas.

Extrapolating from experience in treating both neural and extraneural ependymomas, we employed etoposide as our first-line treatment. Etoposide was demonstrated to be effective in multiple case reports as both single agent and backbone of combination therapy.

Indeed, oral etoposide was an effective agent in this patient leading to prolonged progression-free survival (PFS) as it was the backbone in multiple effective regimens employed. Of note, it demonstrated significant activity even upon rechallenge. Mechanistically, etoposide interacts with topoisomerase-II and prevents the religation of the DNA strand, thereby exerting its tumouricidal effect. It was demonstrated that tumour samples of ependymomas were enriched with topoisomerase-II compared with the control [32]. Also, the level of expression of topoisomerase-II acts as an adverse prognostic factor with correlation with tumour grading and recurrence in ependymoma [32]. Thus, it is possible that etoposide can act upon the upregulated topoisomerase-II enzyme leading to sustained tumouricidal effect.

VIP and TC were both employed upon progression with partial response achieved.

As described previously, the surgical resection of primary peritoneal ependymoma was difficult. This potentially led to a higher rate of local recurrence. In view of the activity of etoposide, adjuvant etoposide-based chemotherapy may be beneficial in reducing recurrence.

### 3.6. Palliative Radiotherapy

Radiotherapy is mainly incorporated as postoperative adjunctive treatment to reduce recurrence of neural ependymoma [33]. Adjuvant radiotherapy is typically delivered from 54 to 59.4 Gy of 1.8 Gy per fraction depending on the site of lesion, grading, and degree of resection [34].

Radiotherapy has been employed for extraneural ependymoma in both adjuvant and palliative settings. Mikami et al. treated a patient with adjuvant pelvic RT for a case of ovarian ependymoma following debulking surgery [14]. Adjuvant RT has been used for cases of sacrococcygeal and mediastinal ependymomas as well [13, 22]. Hirahara et al. employed palliative pelvic RT for a case of ovarian ependymoma; however, clinical response to radiotherapy was not described in the report [15].

Our case clearly demonstrated a remarkable treatment response towards radiotherapy both clinically and radiologically. 28 Gy in 7 daily fractions was sufficient for significant reduction in tumour size as well as palliation of symptoms. It is of note that a palliative dose of biologically effective dose (BED_10_) of 39.2 Gy was already sufficient for significant tumouricidal effect as compared to the BED_10_ of 63.7-70.1 Gy often employed in adjuvant setting. The effect of radiotherapy was also highly durable with local control lasting for 3 years.

### 3.7. Complication of Treatment

It is intriguing that essentially all reported extraneural ependymoma occurs in young females [4, 6, 7, 21]. In situations where aggressive chemotherapy and radiotherapy are indicated, there can be significant complications. Both chemotherapy and radiotherapy would affect fertility and have potential teratogenic effects. In young females with extraneural ependymoma, treatment decisions should consider the fertility wish of the patient. Care also should be taken to avoid the ovaries in the radiation field to preserve the patient's fertility.

Other potential considerations include the risk of secondary malignancy. As patients with extraneural ependymoma are usually young, the cumulative risk of secondary malignancy from treatment is high. Long-term treatment with etoposide is known to be associated with secondary leukaemia in patients with germ cell tumours [35]. Although the risk is hard to estimate, radiation-induced secondary malignancy is also a known complication of radiotherapy [36].

### 3.8. Suggested Treatment Paradigm

In view of potential complications, treatment decisions should be tailor-made while balancing risks, benefits, and disease tempo. In our case, we initiated chemotherapy when the disease load was high and rapid stabilization of disease was needed. Radiation was only given when the patient was suffering from significant local symptoms arising from the tumour. As even the more aggressive form of extraneural ependymoma is still relatively slow growing, we suggest administering chemotherapy for stabilization of disease, followed by drug holiday.

As for choice of chemotherapy, monotherapy with etoposide is effective with a favourable side effect profile. Combination chemotherapy with the etoposide backbone such as VIP/EP/EC/TIP can be considered when rapid disease stabilization is needed, albeit with higher toxicity. Other regimens such as TC can be considered upon disease progression.

Radiotherapy is an effective means for palliation of local symptoms with prolonged durability, but this should be balanced with side effects and risk of secondary malignancy as patients are often young.

## 4. Conclusion

Primary peritoneal ependymoma is a rare disease entity lacking consensus on optimal treatment guidelines. The treatment paradigm was mainly extrapolated from the trial results of recurrent neural ependymoma and scarce case reports. Both chemotherapy and radiotherapy were shown to be effective in the present case. Oral etoposide appears to be particularly useful with other agents such as TC, VIP also demonstrating meaningful activity. Radiotherapy is also an effective means in the treatment of extraneural ependymoma, especially for the palliation of local symptoms. Treatment decisions should be tailor-made, considering patient demographic, tempo of disease progression, and risks and benefits of treatment. Further research and case studies are eagerly awaited to determine the optimal treatment paradigm.

## Figures and Tables

**Table 1:** 

Source	Sex/age	Location	Size	Type	Treatment	Nodal metastasis	Distant metastasis	Follow-up	Outcome
Current case	F/23	Peritoneum	10 cm	Ependymoma, NOS	Surgery, etoposide, cyclophosphamide, VIP, TC, thoracic RT	Yes (mediastinal)	Yes (lung, pleura)	10 years (to date)	AWD, partial response
Verdun and Owen [7]	F/75	Peritoneum	14 cm	Ependymoma, NOS	Surgery	No	No	24 years	Local recurrence after 24 years NED
Yamamoto et al. (2015) [21]	F/23	Peritoneum	Unspecified	Ependymoma, NOS	Surgery	No	No	10 months	AWD
Mogler et al. [3]	F/27	Peritoneum	13 cm	Anaplastic ependymoma	Surgery, imatinib, EC	No	No	Unspecified	Unspecified

The table summarizes the details of reported cases of peritoneal ependymoma. VIP: etoposide/ifosfamide/cisplatin; TC: paclitaxel/carboplatin; EC: etoposide/carboplatin; RT: radiotherapy; AWD: alive with disease; NED: no evidence of disease; NOS: not otherwise specified.

## Abbreviations

VIP: Etoposide/ifosfamide/cisplatin
TC: Paclitaxel/carboplatin
CAP: Cisplatin/aclarubicin/cyclophosphamide
VAC: Vincristine/dactinomycin/cyclophosphamide
EC: Etoposide/carboplatin
PVP: Cisplatin/vinblastine/peplomycin
EP: Etoposide/cisplatin
RT: Radiotherapy
AWD: Alive with disease
NED: No evidence of disease
NOS: Not otherwise specified
CT: Computed tomography
PFS: Progression-free survival.
